# Gut microbiota–host adaptive immune interactions in type 2 diabetes mellitus: mechanisms, disease progression, and microbiota-based therapeutic strategies

**DOI:** 10.3389/fmicb.2026.1934029

**Published:** 2026-09-03

**Authors:** Yi Cheng, Hanxi Zhao, Li Lin, Yuting Chen, Haiyang Lin

**Affiliations:** 1 Department of Endocrinology and Metabolism, The First People's Hospital of Wenling, Wenling, China; 2 Department of Endocrinology and Metabolism, The First Affiliated Hospital of Zhejiang Chinese Medical University, Hangzhou, China

**Keywords:** adaptive immunity, gut microbiota, immunometabolism, microbial metabolites, microbiota-based therapy, type diabetes mellitus

## Abstract

Type 2 diabetes mellitus (T2DM) is a prevalent metabolic disorder whose development and progression are influenced not only by genetic susceptibility, obesity, and lifestyle-related factors but also by gut microbiota dysbiosis, chronic low-grade inflammation, and disruption of immunometabolic homeostasis. The gut microbiota regulates adaptive immune responses through diverse signals, including microbial structural components, metabolites, extracellular vesicles, and other secreted molecules. These microbial-derived signals modulate antigen presentation, T-cell differentiation, B-cell function, and IgA-mediated mucosal immunity, thereby influencing adaptive immune populations such as Th1 cells, Th17 cells, CD8^+^ T cells, and regulatory T cells (Tregs). Conversely, the adaptive immune system can reshape microbial composition and functional outputs by regulating intestinal barrier integrity, mucosal immune homeostasis, and ecological niche stability, forming a dynamic and bidirectional gut microbiota–adaptive immunity interaction network. This reciprocal interaction persists throughout the progression from metabolic risk accumulation and insulin resistance to T2DM onset and diabetic complications, contributing to chronic low-grade inflammation, impaired insulin signaling, and pancreatic β-cell dysfunction. Unlike previous reviews that have primarily focused on gut microbiota dysbiosis, microbial metabolites, or innate immune regulation, this review highlights the bidirectional interactions between the gut microbiota and adaptive immunity as a central framework. We systematically summarize the underlying molecular mechanisms, dynamic disease evolution, and advances in microbiota-targeted therapeutic strategies. In recent years, dietary modulation, probiotics, prebiotics, synbiotics, postbiotics, fecal microbiota transplantation, and engineered microbiome-based therapies have emerged as potential approaches targeting the microbiota–immune axis. Among these strategies, dietary interventions and certain microbiota-based supplements have obtained preliminary clinical support, whereas fecal microbiota transplantation, engineered bacteria, and phage-based therapies remain under active investigation. However, clinical translation of microbiome-targeted therapies is still challenged by interindividual microbiota heterogeneity, insufficient standardization of interventions, uncertain long-term safety, and limited causal evidence. Future efforts should integrate microbial composition, functional metabolic profiles, adaptive immune signatures, and host metabolic states to achieve precise patient stratification and facilitate the development of personalized microbiome-based interventions. This review provides a conceptual framework for understanding immunometabolic mechanisms in T2DM and offers new perspectives for precision therapeutic strategies.

## 1 Introduction

T2DM is one of the most prevalent and burdensome chronic metabolic diseases worldwide, with its prevalence continuing to rise. According to reports from the International Diabetes Federation (IDF), more than 500 million adults globally are currently living with diabetes, with T2DM accounting for the vast majority of cases (Accili et al., 2025). T2DM is primarily characterized by insulin resistance and progressive decline in pancreatic β-cell function, and its development has traditionally been attributed to the combined effects of genetic susceptibility, obesity, and lifestyle-related factors (Huang et al., 2025; Krause and De Vito, 2023). However, accumulating evidence in recent years has demonstrated that T2DM is not merely a metabolic disorder but also a complex disease accompanied by chronic low-grade inflammation and immunometabolic dysregulation (Chen Y. et al., 2026). Persistent immune activation not only contributes to the development of insulin resistance but also promotes β-cell dysfunction, thereby accelerating disease progression and the development of diabetes-associated complications (Berbudi et al., 2025; Papareddy and Herwald, 2025).

The gut microbiota represents a fundamental component of host physiology, playing essential roles in nutrient metabolism, intestinal barrier maintenance, and immune homeostasis (Ding et al., 2025). Increasing evidence indicates that individuals with T2DM exhibit varying degrees of gut microbial and functional alterations, including changes in microbial composition, disrupted metabolic capacity, and imbalanced microbial-derived signaling (Rondanelli et al., 2025; Han et al., 2026). Within the interface between gut microbial perturbations and host immunometabolic dysfunction, adaptive immunity may serve as a critical regulatory bridge. Unlike innate immunity, which primarily mediates early pattern recognition and acute inflammatory initiation, adaptive immunity possesses antigen specificity, immunological memory, and long-term regulatory capacity. Dysregulated remodeling of adaptive immune responses may therefore represent a key mechanism linking transient microbial disturbances to persistent chronic low-grade inflammation in T2DM. Accordingly, adaptive immunity provides a new conceptual framework for understanding how microbial alterations are translated into sustained immunometabolic abnormalities.

Gut microbiota-derived structural components, metabolites, extracellular vesicles, and other microbial-associated signals can modulate adaptive immune responses through mechanisms involving antigen presentation and immunometabolic regulation, thereby influencing T-cell differentiation, B-cell function, and IgA-mediated mucosal immune homeostasis (Arifuzzaman et al., 2024; Takeuchi et al., 2024; Fu et al., 2023). Conversely, alterations in adaptive immune status can reshape gut microbial composition, spatial organization, and ecological stability by regulating intestinal barrier integrity, the mucus layer, antimicrobial peptide expression, and IgA-mediated mucosal defense (Wang Z. et al., 2026; Kwon et al., 2026). The continuous bidirectional regulation between these two systems may collectively contribute to the initiation and maintenance of chronic low-grade inflammation, insulin resistance, and immunometabolic imbalance in T2DM.

Although the relationship between the gut microbiota and T2DM has been extensively investigated, previous reviews have primarily focused on microbial dysbiosis, microbial metabolites, intestinal barrier disruption, and innate immune regulation. In contrast, the bidirectional interactions between microbiota-derived signals and adaptive immune responses remain insufficiently integrated. In particular, the regulatory relationships between distinct microbial signals, including structural components, metabolites, and extracellular vesicles, and specific adaptive immune subsets, as well as the dynamic evolution of this microbiota–immune interaction network across different stages of T2DM, have not been comprehensively summarized.

**Figure 1:**
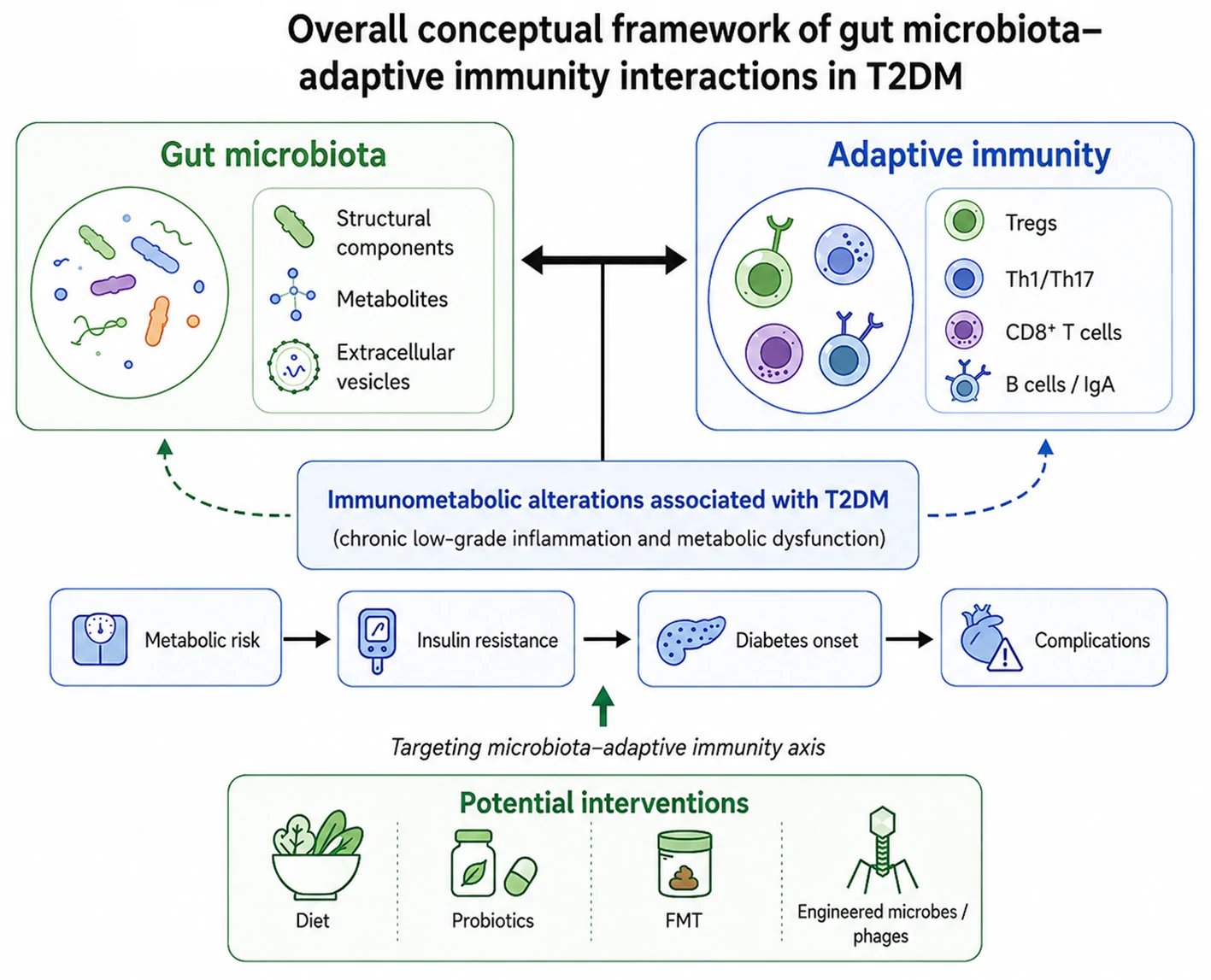
Overall conceptual framework of gut microbiota–adaptive immunity interactions in T2DM. Gut microbiota-derived structural components, metabolites, and extracellular vesicles interact bidirectionally with adaptive immune components, including Tregs, Th1/Th17 cells, CD8^+^ T cells, and B cell/IgA responses. Disruption of this crosstalk contributes to chronic low-grade inflammation and metabolic dysfunction and is associated with progression from metabolic risk and insulin resistance to diabetes onset and complications. Microbiota-targeted interventions, including diet, probiotics, FMT, and engineered microbes or phages, may help restore microbiota–adaptive immune homeostasis and improve immunometabolic outcomes.

Therefore, this review focuses on the gut microbiota–adaptive immunity interaction axis as a central framework. We systematically discuss the molecular mechanisms by which microbial-derived signals regulate adaptive immune responses, analyze the dynamic alterations of microbiota–immune interactions throughout different stages of T2DM progression, and summarize microbiota-targeted intervention strategies targeting the gut microbiota–adaptive immunity axis together with their translational challenges. This review aims to provide new insights into the immunometabolic mechanisms underlying T2DM and facilitate the development of precision microbiome-based therapeutic strategies. The overall conceptual framework and major components of this review are summarized in Figure 1.

## 2 Gut microbiota alterations and adaptive immune dysregulation in T2DM

During the development and progression of T2DM, alterations in the gut microbiota and adaptive immune dysregulation frequently coexist and collectively contribute to the immunometabolic imbalance underlying the disease. Compared with healthy individuals, patients with T2DM exhibit varying degrees of alterations in gut microbial composition and functional capacity (Han et al., 2026). At the taxonomic level, several human studies have consistently reported reductions in short-chain fatty acid (SCFA)-producing bacteria, including *Faecalibacterium, Roseburia*, and *Eubacterium*, accompanied by relative enrichment of certain members of the *Enterobacteriaceae* family and other opportunistic pathogenic bacteria (Chen Y. et al., 2026). These findings suggest that T2DM-associated microbial dysbiosis involves not only the loss of beneficial microbial functions but also a potential increase in pro-inflammatory microbial features. However, specific microbial alterations are not entirely consistent across studies, likely due to differences in population characteristics, dietary patterns, medication exposure, disease stage, and analytical methodologies. Moreover, geographic background, obesity status, disease duration, sequencing platforms, and bioinformatic pipelines may further contribute to variations among microbiome studies. Importantly, current human cohort studies primarily demonstrate associations between microbial alterations and metabolic or immune abnormalities in T2DM, whereas animal models and microbiota intervention studies provide additional mechanistic insights and potential causal evidence. Given the differences in dietary exposure, microbial composition, and immune environments between animal models and humans, these findings require further validation through longitudinal human studies (Chen Y. et al., 2026; Han et al., 2026).

Gut microbial dysbiosis can substantially influence host immune homeostasis. Reduced abundance of SCFA-producing bacteria may impair intestinal barrier protection and the maintenance of immune tolerance (Yu et al., 2025), whereas alterations in bile acid and tryptophan metabolism may modify immune cell differentiation and inflammatory response thresholds (Yoon et al., 2023; Arifuzzaman et al., 2024). Increasing evidence further indicates that SCFAs contribute to the differentiation and stability of Foxp3^+^ Tregs and influence B-cell responses and IgA-mediated mucosal immunity. Meanwhile, bile acid- and tryptophan-derived metabolites can regulate Treg/Th17 balance and other adaptive immune responses through pathways involving farnesoid X receptor (FXR), Takeda G protein-coupled bile acid receptor 1 (TGR5), and aryl hydrocarbon receptor (AhR) (Arifuzzaman et al., 2024; Takeuchi et al., 2024; Fu et al., 2023). These findings suggest that specific microbial metabolites may serve as critical molecular intermediates linking microbial functional alterations to adaptive immune dysregulation. In addition, high-fat diets, obesity, and hyperglycemic conditions can compromise intestinal barrier integrity, increasing exposure to microbial structural components, metabolites, and microbial antigens, thereby triggering low-grade immune activation (Zheng et al., 2025; Tang and de Vos, 2025). Such intestinal immune disturbances may subsequently influence metabolic organs, including adipose tissue, liver, skeletal muscle, and pancreatic islets, through inflammatory mediator release, immune cell trafficking, and altered metabolic signaling, thereby establishing an inflammatory environment that promotes insulin resistance and β-cell dysfunction (Man et al., 2022; Yan, 2024; Shaikh et al., 2024).

Adaptive immune dysregulation represents a critical component of immunometabolic imbalance in T2DM. Under conditions of obesity and insulin resistance, T-cell subsets undergo functional remodeling, characterized by enhanced pro-inflammatory Th1 and Th17 responses and impaired regulatory T-cell activity, resulting in reduced immune tolerance and persistent low-grade inflammation (Zi et al., 2022; Shirakawa and Sano, 2023; Lin M. et al., 2026). CD8^+^ T cells may also contribute to the amplification of adipose tissue inflammation and metabolic tissue injury (Nishimura et al., 2009). Beyond T cells, B cells participate in immunometabolic regulation through antigen presentation, inflammatory cytokine production, and antibody-mediated responses (Rastogi et al., 2022). Meanwhile, intestinal IgA responses are closely associated with microbial spatial organization, mucosal barrier integrity, and the regulation of commensal microbial populations (Di Sabatino et al., 2023; Takada, 2025).

**Figure 2:**
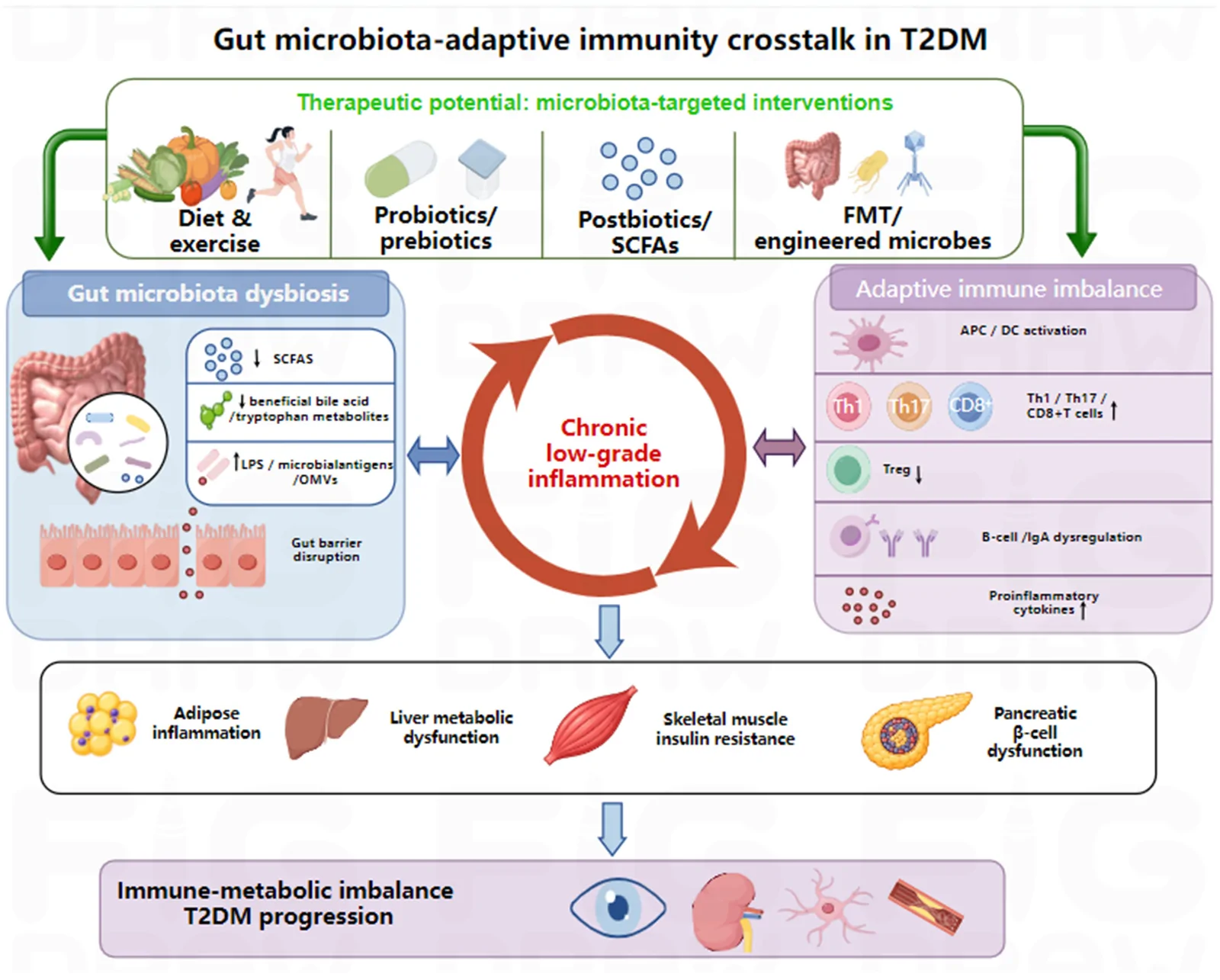
Bidirectional interaction between gut microbiota dysbiosis and adaptive immune imbalance in type 2 diabetes mellitus. Gut microbiota dysbiosis contributes to adaptive immune disturbance through altered microbial-derived signals, including reduced beneficial metabolites such as short-chain fatty acids, bile acid derivatives, and tryptophan metabolites, together with increased exposure to inflammatory microbial components, including lipopolysaccharide, microbial antigens, and extracellular vesicles. These alterations promote pro-inflammatory T-cell responses characterized by enhanced Th1, Th17, and CD8^+^ T-cell activity, accompanied by impaired regulatory T-cell and B cell/IgA-mediated immune tolerance. Conversely, adaptive immune imbalance further disrupts intestinal barrier integrity and microbial ecological stability, establishing a self-amplifying feedback loop. This microbiota–adaptive immunity crosstalk contributes to chronic low-grade inflammation, metabolic tissue dysfunction, and progressive immunometabolic imbalance in T2DM.

Therefore, alterations in microbial composition and function can influence T-cell responses, B-cell activity, and IgA-mediated immunity through antigen stimulation, metabolite signaling, and barrier disruption. Conversely, adaptive immune abnormalities may impair mucosal barrier repair and microbial ecological stability, thereby further sustaining microbial dysbiosis. Current human evidence remains insufficient to determine whether microbiota alterations represent initiating drivers of T2DM development or secondary consequences of metabolic disturbances, including obesity, hyperglycemia, medication exposure, and chronic inflammation. Instead, these processes likely form a self-amplifying feedback loop that continuously imposes inflammatory and metabolic stress during T2DM progression (Figure 2).

## 3 Molecular mechanisms underlying gut microbiota–adaptive immunity crosstalk

### 3.1 Regulation of adaptive immunity by the gut microbiota

#### 3.1.1 Microbial structural components-mediated regulation of adaptive immunity

Microbial structural components represent important sources of signals through which the host recognizes intestinal microorganisms and initiates adaptive immune responses. These components include lipopolysaccharide (LPS), lipoteichoic acid (LTA), flagellin, peptidoglycan, and microbial-derived nucleic acids (Vilela Rodrigues et al., 2026). Such microbe-associated molecular patterns (MAMPs) are initially sensed by pattern recognition receptors (PRRs) expressed on antigen-presenting cells (APCs), including dendritic cells and macrophages. Among these signals, LPS primarily activates immune signaling through Toll-like receptor 4 (TLR4), whereas LTA and certain peptidoglycan components are recognized by TLR2- and NOD-like receptor (NLR)-associated pathways. Flagellin activates TLR5, while microbial nucleic acids participate in immune recognition through receptors such as TLR9 (Charoensaensuk et al., 2024; Luo et al., 2025; Xu et al., 2025; Li et al., 2024). These sensing events subsequently activate downstream signaling pathways, including MyD88/TRIF, nuclear factor-κB (NF-κB), and mitogen-activated protein kinase (MAPK) pathways, promoting APC maturation, antigen processing and presentation, and the expression of costimulatory molecules such as CD80/CD86 and inflammatory cytokines. Through these processes, microbial pattern-recognition signals are converted into adaptive immune responses.

The cytokine milieu established by APCs further determines the differentiation fate of naïve CD4^+^ T cells. For example, IL-12-associated signaling promotes STAT4 and T-bet activation, driving Th1 differentiation, whereas IL-6, IL-23, and related inflammatory signals facilitate Th17 responses through STAT3 and RORγt activation. In contrast, IL-10, transforming growth factor-β (TGF-β), and tolerogenic APC environments favor the generation of Foxp3^+^ Tregs and the maintenance of immune tolerance (Murugesan et al., 2025; Schnell et al., 2023; van de Vyver, 2023). In the context of T2DM, intestinal barrier disruption and microbial dysbiosis may increase persistent exposure to pro-inflammatory microbial structural components, promoting the formation of inflammatory costimulatory and cytokine environments by APCs. This process favors Th1 and Th17 effector responses while weakening Treg-mediated immune tolerance (Murugesan et al., 2025; Schnell et al., 2023; van de Vyver, 2023). Conversely, structural signals derived from commensal microorganisms, such as capsular polysaccharides and exopolysaccharides, can promote immunoregulatory tolerance, enhance Treg differentiation, support IgA-mediated mucosal immune homeostasis, and restrict excessive inflammation and uncontrolled microbial expansion (Yang et al., 2023; Zhao T. et al., 2025; Ding et al., 2025). Therefore, in T2DM, enhanced pro-inflammatory microbial structural signaling together with insufficient tolerogenic microbial cues may collectively contribute to disruption of the Treg/Th17 balance, abnormal B-cell responses, and impaired IgA homeostasis (Yang H.Y. et al., 2025; Wang H.W. et al., 2023; Li J. et al., 2026).

Importantly, the immunological effects of microbial structural components are highly dependent on exposure levels and biological context and cannot be simply categorized as either pro-inflammatory or anti-inflammatory. Their effects are influenced by multiple factors, including microbial origin, structural characteristics, exposure dose and duration, intestinal barrier integrity, and the metabolic and immune status of the host. For example, transient or preconditioning LPS exposure has been shown in certain experimental models to induce tolerance-like immune reprogramming, whereas sustained or excessive exposure to microbial-associated molecules, particularly under conditions of barrier dysfunction and metabolic inflammation, is more likely to maintain APC activation and pro-inflammatory T-cell responses (Vilela Rodrigues et al., 2026; Charoensaensuk et al., 2024). It should also be emphasized that current mechanistic evidence regarding TLR/NOD-mediated recognition, APC maturation, and specific T-cell differentiation pathways is largely derived from *in vitro* studies and animal models, including murine systems (Charoensaensuk et al., 2024; Luo et al., 2025; Xu et al., 2025; Li et al., 2024). Human studies in T2DM have mainly provided associative evidence linking microbial translocation, circulating microbial-derived signals, inflammatory status, and immune abnormalities. Direct validation of the complete mechanistic cascade from microbial structural components to PRR activation, APC programming, and specific T-cell differentiation in humans remains limited.

#### 3.1.2 Microbial metabolite-mediated regulation of adaptive immunity

Unlike microbial structural components, which primarily initiate adaptive immune responses through antigen recognition, microbial metabolites function as major mediators of microbial functional outputs and continuously participate in the regulation of host immune homeostasis. SCFAs, bile acids, tryptophan-derived metabolites, branched-chain amino acid (BCAA)-related metabolites, trimethylamine N-oxide (TMAO), polyamines, and other metabolites generated through microbial fermentation and metabolism can regulate T-cell differentiation, B-cell function, and IgA-mediated mucosal immunity through diverse signaling pathways, including GPCRs, AhR, FXR/TGR5, and mTOR, thereby shaping adaptive immune homeostasis.

SCFAs represent a major class of microbial metabolites linking gut microbial metabolism with adaptive immune regulation. In addition to signaling through GPCRs, butyrate and propionate can inhibit histone deacetylase (HDAC) activity, increase histone acetylation within Foxp3 regulatory regions, enhance Foxp3 transcription, and promote Treg differentiation and stability (Du et al., 2022; Zhang et al., 2024; Wei et al., 2024; Shemtov et al., 2023). This epigenetic regulatory mechanism contributes to the maintenance of immune tolerance and restricts excessive pro-inflammatory T-cell responses, including Th17 activation.

Bile acids exert more complex receptor-dependent immunoregulatory effects. As a nuclear receptor, FXR regulates transcriptional programs involved in bile acid synthesis, lipid and glucose metabolism, and inflammatory responses upon bile acid binding. In contrast, TGR5, a membrane-associated GPCR, modulates immune cell inflammatory responses and intestinal endocrine functions through cyclic adenosine monophosphate (cAMP)-dependent signaling. Together, FXR and TGR5 serve as key signaling interfaces connecting bile acid metabolism with host immunometabolic regulation and may influence Treg/Th17 balance by altering cytokine environments and cellular metabolic states (Chen H. et al., 2026; Yue et al., 2026; Lin B.S.et al., 2026; Li D. et al., 2023; Liu Z. et al., 2026; Li et al., 2025c). Furthermore, indole derivatives generated from microbial tryptophan metabolism can activate AhR-associated pathways to promote Treg responses and IL-22-related immune functions, whereas metabolites such as polyamines, lactate, and succinate may exert context-dependent regulatory effects depending on local concentrations, tissue environments, and immune cell metabolic states (Chen et al., 2023; Kang et al., 2025; Shen et al., 2022; Shaheen et al., 2025; Schütz et al., 2025; Zhang et al., 2022; Stone and Williams, 2023; Ogbechi et al., 2022; Wang et al., 2025a; Zhu et al., 2024; Alsaleh et al., 2026; Gu et al., 2026; Yang H. et al., 2025; Bündgen et al., 2025; Ocegueda et al., 2026; Wang et al., 2025d; Li and Tian, 2026; Wang X. et al., 2026).

Importantly, the biological effects of microbial metabolites are not always unidirectional. TMAO represents one of the most debated examples. Several clinical and experimental studies have reported that elevated circulating TMAO levels are associated with metabolic inflammation, endothelial dysfunction, and increased cardiovascular risk in diabetes, potentially accompanied by enhanced pro-inflammatory immune responses (Wang et al., 2025a; Zhu et al., 2024). However, circulating TMAO concentrations are simultaneously influenced by multiple factors, including dietary choline and carnitine intake, gut microbial composition, hepatic oxidation capacity, and renal clearance. Therefore, increased TMAO levels may reflect alterations in microbial metabolism as well as secondary changes caused by host metabolic disturbances or renal dysfunction. Currently, TMAO should not be considered a direct and independent driver of adaptive immune inflammation, and evidence supporting its direct effects on adaptive immune subsets, including Th1, Th17, and B cells, remains less established than that for metabolites such as SCFAs. Similarly, the immunological effects of specific bile acids, organic acids, and tryptophan-derived metabolites are also highly dependent on concentration, tissue context, and disease stage.

The biological significance of microbial metabolites extends beyond immune regulation and is deeply integrated with systemic metabolic networks. SCFAs influence intestinal hormone secretion, energy utilization, and glucose and lipid metabolism in the liver, adipose tissue, and skeletal muscle. Bile acids regulate hepatic glucose and lipid metabolism, energy expenditure, and incretin signaling through FXR- and TGR5-dependent pathways. Dysregulated BCAA metabolism has been linked to enhanced mTOR signaling and insulin resistance, whereas TMAO and certain organic acids may influence vascular function, oxidative stress, and cardiovascular metabolic risk. Therefore, microbial metabolites are more likely to function as shared signaling nodes connecting gut microbial activity, adaptive immunity, and systemic metabolic states rather than merely serving as local immune mediators.

In T2DM, altered levels of multiple microbiota-associated metabolites have been detected in blood, fecal, or urinary samples and have been associated with insulin resistance, glycemic control, inflammatory status, and complication risks. However, these metabolic signatures currently remain largely at the stage of candidate biomarkers, and no single microbial metabolite has been independently validated through multicenter studies for T2DM diagnosis, patient stratification, or prognosis prediction. Their clinical applicability is further limited by variations in dietary intake, medication exposure, renal function, host metabolic status, sample type, and analytical platforms. Therefore, future efforts may benefit from integrating multiple microbial metabolites with microbial functional profiles, adaptive immune signatures, and conventional metabolic parameters to establish multidimensional biomarker systems rather than relying on individual metabolites. Overall, microbial metabolites represent not only critical mediators linking microbial functional alterations to adaptive immune remodeling but also key nodes connecting local immune regulation with systemic metabolic dysfunction. The major adaptive immune targets of microbial metabolites and their potential immunometabolic implications in T2DM are summarized in Table 1.

**Table 1:** Major microbial-derived metabolites regulating adaptive immunity and their potential immunometabolic implications in T2DM.

Category	Microbial metabolite	Major adaptive immune targets	Major effects on adaptive immunity	Potential immunometabolic implications in T2DM	References
Short-chain fatty acids	Acetate	Tregs; B cells	Enhances Treg function and promotes IgA production	Maintains mucosal immune homeostasis, strengthens IgA-mediated barrier protection, and may reduce microbial antigen exposure and low-grade inflammation	Bergstrom and Xia, 2022; López-Fandiño, 2025
Short-chain fatty acids	Propionate	Tregs; Th17 cells	Promotes Treg differentiation and suppresses excessive Th17 polarization	Enhances immune tolerance, limits Th17-associated inflammation, and may alleviate adipose tissue inflammation and insulin resistance	Du et al., 2022; Zhang et al., 2024
Short-chain fatty acids	Butyrate	Tregs; IgA-producing plasma cells	Induces Foxp3^+^ Treg differentiation and enhances IgA responses	Strengthens intestinal barrier integrity and IgA-mediated immunity, potentially reducing metabolic endotoxemia, improving insulin sensitivity, and preserving β-cell function	Wei et al., 2024; Shemtov et al., 2023
Bile acids	Primary bile acids	Tregs; B cells	Regulates Treg/Th17 balance through FXR- and TGR5-dependent pathways	Modulates glucose and lipid metabolism as well as immune inflammation, potentially affecting insulin sensitivity, hepatic metabolism, and intestinal immune homeostasis	Chen H. et al., 2026; Yue et al., 2026
Bile acids	Deoxycholic acid (DCA)	Th17 cells; Tregs	Modulates Th17-associated inflammatory responses in a context-dependent manner	May influence Th17 inflammatory thresholds, intestinal barrier stability, and metabolic inflammation	Lin B.S.et al., 2026; Li D. et al., 2023
Bile acids	Lithocholic acid (LCA)	Tregs; Th17 cells	Promotes Treg differentiation and restrains Th17-associated inflammatory responses	Maintains immune tolerance, reduces chronic low-grade inflammation, and may improve metabolic homeostasis	Liu Z. et al., 2026; Li et al., 2025c
Tryptophan metabolites	Indole derivatives	Tregs; IL-22-associated T cells	Activates AhR signaling, promotes Treg and IL-22-related responses, and limits excessive Th17 activation	Maintains intestinal barrier integrity, reduces microbial translocation, alleviates systemic inflammation, and may improve insulin resistance	Chen et al., 2023; Kang et al., 2025
Tryptophan metabolites	Indole-3-acetic acid (IAA)	Tregs; Th17 cells	Enhances immune tolerance and regulates inflammatory responses	Improves the intestinal inflammatory microenvironment and reduces inflammatory burden in metabolic tissues	Shen et al., 2022; Shaheen et al., 2025
Tryptophan metabolites	Indole-3-propionic acid (IPA)	Tregs; barrier-associated T cells	Stabilizes Treg function and supports barrier-protective immune responses	Reduces metabolic inflammation, improves insulin sensitivity, and may delay T2DM progression	Schütz et al., 2025; Zhang et al., 2022
Tryptophan metabolites	Kynurenine	Tregs; effector T cells	Regulates T-cell differentiation through the AhR/IDO axis, promoting immunosuppressive or tolerogenic states	May influence chronic inflammation, immune tolerance balance, and immunoregulatory states in T2DM	Stone and Williams, 2023; Ogbechi et al., 2022
Choline metabolites	Trimethylamine N-oxide (TMAO)	Th1 cells; Th17 cells; B cells	Indirectly enhances pro-inflammatory immune responses and promotes inflammatory cytokine production and adaptive immune deviation	May aggravate metabolic inflammation, endothelial dysfunction, and increase the risk of diabetic cardiovascular complications	Wang et al., 2025c; Zhu et al., 2024
Polyamines	Spermidine	Tregs; B cells	Promotes Treg stability and regulates antibody responses and autophagy-related immune processes	May alleviate chronic inflammation, improve metabolic homeostasis, and preserve tissue function	Alsaleh et al., 2026; Gu et al., 2026
Polyamines	Spermine	Tregs; effector T cells	Restrains excessive immune activation and regulates T-cell effector functions	May reduce inflammatory burden and metabolic stress while maintaining immune homeostasis	Yang H. et al., 2025; Bündgen et al., 2025
Organic acids	Succinate	Th17 cells; effector T cells	Promotes pro-inflammatory immune responses and enhances Th17-associated inflammation	May exacerbate chronic low-grade inflammation, insulin resistance, and metabolic tissue injury	Ocegueda et al., 2026; Wang et al., 2025d
Organic acids	Lactate	Tregs; effector T cells	Regulates T-cell metabolic states and local immune thresholds in a context-dependent manner	May influence intestinal immune homeostasis, inflammatory persistence, and metabolic adaptation	Li and Tian, 2026; Wang X. et al., 2026

#### 3.1.3 Microbial extracellular vesicle- and secreted molecule-mediated regulation of adaptive immunity

Gut microorganisms can regulate adaptive immune responses through extracellular vesicles (EVs), outer membrane vesicles (OMVs), secreted proteins, microbial-derived nucleic acids, extracellular ATP, and other bioactive molecules (Xia et al., 2025; Zhu Y. et al., 2026). These molecules can carry diverse functional components, traverse the mucus layer, or be transported across epithelial cells and microfold cells, after which they are internalized by dendritic cells, macrophages, and B cells. Through these processes, microbial vesicles and secreted factors can influence antigen presentation, costimulatory signaling, and cytokine environments, thereby shaping adaptive immune responses (Napiórkowska-Baran et al., 2025; Zhang H. et al., 2025; Zhang Y. et al., 2025).

The biogenesis of microbial EVs is regulated by multiple factors, including bacterial envelope architecture, membrane lipid composition, cell wall remodeling, and environmental stress conditions. OMVs produced by Gram-negative bacteria are primarily generated through localized outward curvature and budding of the outer membrane, followed by detachment from the bacterial surface. Their release can be influenced by weakened outer membrane–peptidoglycan interactions, redistribution of lipopolysaccharides and phospholipids, accumulation of periplasmic components, and envelope stress responses. In addition, some Gram-negative bacteria may generate distinct membrane vesicle populations through cell lysis or remodeling of double-membrane structures (De Langhe et al., 2024). In contrast, Gram-positive bacteria lack an outer membrane, and their EVs must traverse a thick peptidoglycan layer, a process that may involve membrane budding, localized cell wall degradation, and cell wall remodeling (Zhao X. et al., 2025; Welsh et al., 2024).

OMVs derived from pro-inflammatory microbial communities can enhance dendritic cell maturation, increase the expression of CD80/CD86 and inflammatory cytokines, and promote Th1 and Th17 responses through IL-6-, IL-12-, and IL-23-associated signaling pathways (Polonio et al., 2025; Zhang Y.F et al., 2025). Some OMVs can also transport microbial DNA, activate stimulator of interferon genes (STING)-related pathways, and enhance T-cell activation responses (Zhang J. et al., 2026). In addition, extracellular ATP may promote the release of inflammatory mediators through purinergic receptor signaling, thereby indirectly reinforcing pro-inflammatory T-cell responses (Li W. et al., 2026).

Conversely, EVs derived from certain commensal or probiotic microorganisms exhibit immunoregulatory properties. Polysaccharides and other functional molecules carried by these vesicles can induce tolerogenic dendritic cell phenotypes, enhance anti-inflammatory signaling such as IL-10 production, promote Treg differentiation, and support IgA-mediated mucosal immune homeostasis (Wang et al., 2025b; Li et al., 2025a). These findings indicate that microbial vesicles are not merely inflammatory carriers but represent important communication systems through which the microbiota exchanges biological information with the host immune system. Increased production of vesicles and secreted molecules from pro-inflammatory microbial populations may contribute to sustained antigen presentation, dysregulated T-cell polarization, and impaired IgA regulation, thereby further promoting gut microbial instability and immunometabolic dysfunction.

Microbial EVs have recently attracted attention as postbiotic-like immunomodulatory agents and biological delivery platforms. EVs derived from beneficial microorganisms may provide potential advantages by delivering functional molecules to host tissues without requiring persistent colonization by live bacteria (Polonio et al., 2025). Furthermore, engineered OMVs have been explored as vehicles for delivering antigens, therapeutic compounds, and immunomodulatory molecules, demonstrating potential applications in experimental models of infection, inflammatory diseases, and cancer immunotherapy (Humaira et al., 2024; Wang et al., 2025c). In metabolic diseases, emerging evidence suggests that gut microbiota-derived EVs may influence obesity- and diabetes-associated phenotypes by regulating intestinal barrier integrity, inflammation, and glucose metabolism (Díez-Sainz et al., 2022). Notably, EVs derived from *Akkermansia muciniphila* have been shown to improve intestinal barrier function, glucose tolerance, and metabolic abnormalities in high-fat diet-fed mice. In addition, early human observational studies have reported reduced abundance or altered EV-associated signals in fecal samples from individuals with T2DM compared with healthy controls (Chelakkot et al., 2018). However, these findings are primarily derived from cell-based studies, animal models, and limited human observational investigations rather than randomized intervention trials directly targeting microbial EVs in T2DM.

Further investigation of microbial EVs is also challenged by methodological heterogeneity in their isolation and characterization. Current approaches include differential ultracentrifugation, density gradient centrifugation, ultrafiltration, and size-exclusion chromatography; however, these techniques differ substantially in recovery efficiency, purity, particle-size selection, and preservation of vesicle bioactivity (De Langhe et al., 2024; Welsh et al., 2024). Particularly in complex biological samples such as feces and blood, microbial EVs coexist with host-derived EVs, lipoproteins, protein aggregates, free nucleic acids, bacteriophages, and other non-vesicular particles, making specific enrichment and source identification challenging. Moreover, EV compositions vary considerably among bacterial species, and universal molecular markers applicable to all microbial EVs have not yet been established. Therefore, reliable identification of microbial EVs requires integration of multiple approaches, including nanoparticle tracking analysis, electron microscopy, proteomic and lipidomic profiling, nucleic acid analysis, and functional validation, rather than relying solely on particle size or morphological characteristics.

From the perspective ofcurrent evidence levels, our understanding of microbial EV biogenesis, host cellular uptake, antigen delivery, and immune regulation remains largely derived from bacterial culture systems, *in vitro* experiments, and animal models. Human studies have mainly reported observational differences in microbial EV composition, abundance, or associated molecular signatures under different disease conditions. In contrast, interventional evidence demonstrating that specific microorganism-derived EVs directly regulate adaptive immunity and improve clinical outcomes in T2DM remains extremely limited.

### 3.2 Reciprocal regulation of the gut microbiota by adaptive immunity

Adaptive immunity is not merely a target of gut microbiota regulation but can also reciprocally influence microbial composition, spatial distribution, and functional outputs by shaping the intestinal ecological niche. Tregs maintain epithelial barrier integrity, mucus layer stability, and immune tolerance through the production of regulatory mediators such as IL-10 and TGF-β, thereby influencing the colonization and persistence of commensal microorganisms (Okumura and Takeda, 2024; Cui et al., 2026). Conversely, enhanced Th1 responses can disrupt epithelial tight junction integrity through inflammatory cytokines such as IFN-γ, increasing intestinal permeability and facilitating greater exposure of microbial components to mucosal immune surveillance (Zhang Y. et al., 2026). Dysregulated Th17-associated responses may further remodel the microbial niche by altering IL-17-, IL-22-, and downstream epithelial repair pathways, thereby affecting mucus layer organization and epithelial barrier renewal (An et al., 2026; Shen et al., 2026).

These adaptive immune responses are primarily organized and maintained within gut-associated lymphoid tissue, where Peyer's patches serve as critical sites linking microbial antigen sampling with mucosal adaptive immune responses. Microbial antigens transported into Peyer's patches through M cells and other antigen sampling pathways can be recognized by dendritic cells and B cells, promoting either T cell-dependent or T cell-independent IgA responses. Activated IgA? B cells subsequently migrate to the intestinal lamina propria and differentiate into IgA-secreting plasma cells, converting local antigen recognition into sustained mucosal immune regulation (Okumura and Takeda, 2024).

T-cell-derived cytokines also influence microbial composition through the regulation of antimicrobial peptides and mucosal secretory factors. IL-22 acts on intestinal epithelial cells and Paneth cells to promote the expression of barrier- and antimicrobial-associated molecules, including MUC2, regenerating islet-derived protein IIIγ (RegIIIγ), and β-defensins, thereby limiting bacterial adhesion and uncontrolled expansion (Wang S. et al., 2026; Qi et al., 2025). A properly regulated IL-17/IL-22 axis contributes to epithelial repair and microbial ecosystem stability (Xie and Guo, 2026). However, under chronic inflammatory conditions, persistent activation or functional imbalance of this axis may alter antimicrobial peptide profiles, disrupt mucus layer architecture, and reshape local ecological pressures, allowing certain opportunistic pathogens to gain competitive advantages.

The B cell–IgA axis represents another central mechanism through which adaptive immunity regulates the gut microbiota. Intestinal B cells undergo IgA class switching and differentiate into IgA-secreting plasma cells in response to both T cell-dependent and T cell-independent signals (Reboldi et al., 2016). Secretory IgA selectively coats specific commensal or potentially pathogenic microorganisms, limiting their mucosal attachment, invasion, and excessive expansion through immune exclusion, while reducing the translocation of microbial antigens across the intestinal barrier into mucosal tissues (Gao et al., 2024; Zhang Y. et al., 2026). IgA coating can also modify bacterial interactions with the mucus layer and epithelial surface and influence bacterial motility, virulence traits, and metabolic activity, thereby regulating microbial colonization competition and ecological niche occupation (Mori and Goto, 2026).

Importantly, current evidence does not support a universal temporal sequence between adaptive immune dysfunction and microbial alterations. Microbial dysbiosis can induce adaptive immune remodeling through antigen exposure, metabolite signaling, and barrier disruption, whereas abnormalities in T-cell responses and B-cell/IgA functions can reciprocally reshape epithelial barriers, antimicrobial environments, and microbial ecological niches. Therefore, in T2DM, these processes are more likely to constitute a mutually reinforcing bidirectional feedback loop rather than a fixed unidirectional causal pathway. The precise temporal relationship between microbial alterations and adaptive immune dysfunction requires further clarification through longitudinal human studies.

## 4 Gut microbiota–adaptive immunity interactions during T2DM progression

**Figure 3:**
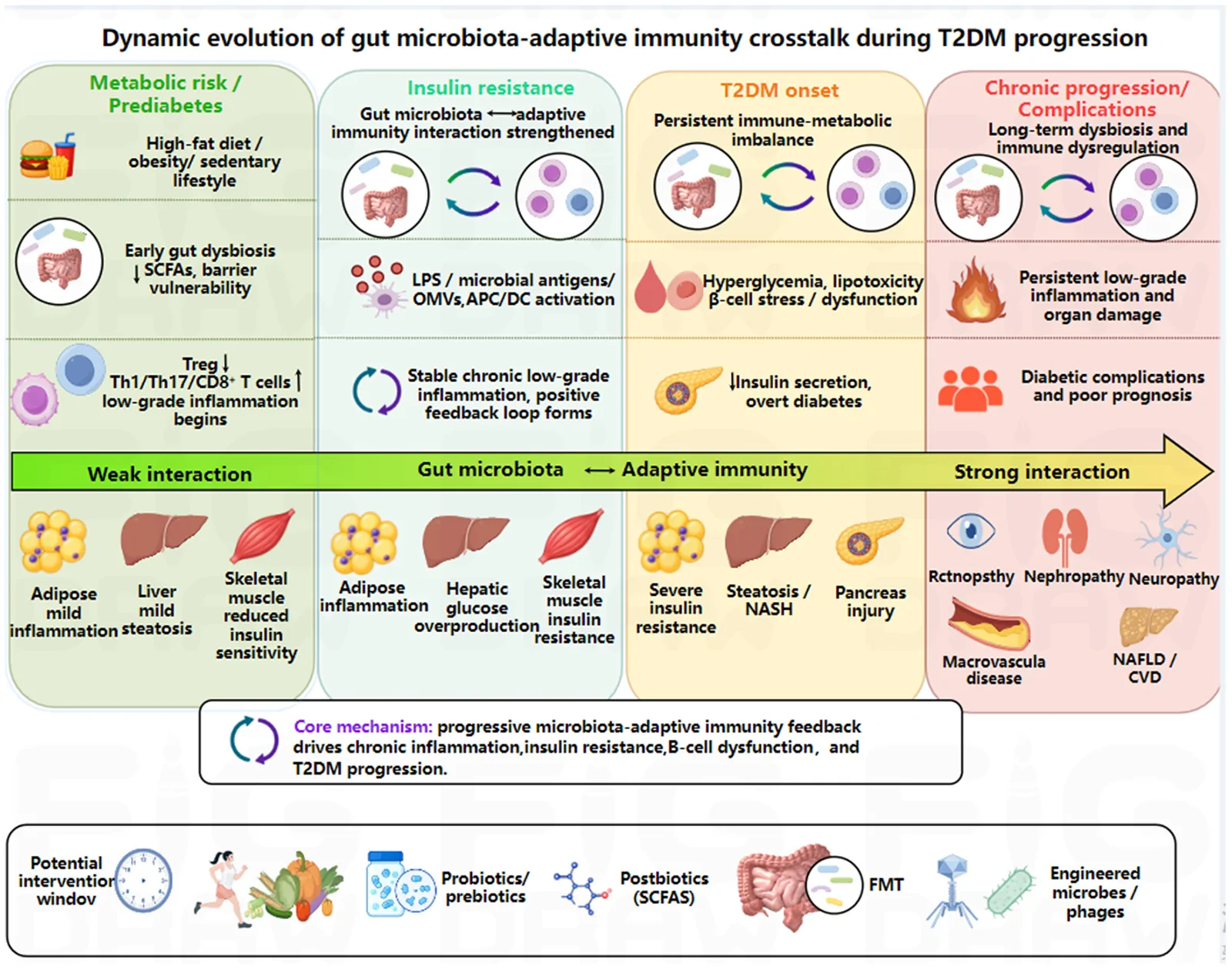
Dynamic evolution of gut microbiota–adaptive immunity crosstalk during type 2 diabetes mellitus progression. The interaction between gut microbiota and adaptive immunity undergoes progressive remodeling across different stages of T2DM development. During the metabolic risk stage, early microbial dysbiosis, impaired intestinal barrier function, and reduced immune tolerance create a permissive inflammatory environment. With the development of insulin resistance, microbial-derived inflammatory signals and adaptive immune activation establish a persistent positive feedback loop, promoting metabolic tissue inflammation. During established T2DM, sustained microbiota–immune imbalance cooperates with hyperglycemia, lipotoxicity, and β-cell stress to accelerate disease progression. In chronic progression and complication stages, persistent low-grade inflammation and immune dysregulation contribute to multi-organ injury and diabetic complications. The gradual strengthening of microbiota–adaptive immunity interactions represents a continuous driver of T2DM progression.

The development of T2DM represents a continuous process involving coordinated alterations in metabolic homeostasis, gut microbial ecology, and adaptive immune remodeling. Current evidence suggests that gut microbiota–adaptive immunity interactions may contribute to multiple stages of T2DM progression, including metabolic risk accumulation, establishment of insulin resistance, diabetes onset, and the development of chronic complications (Figure 3). The characteristics and dominant features of microbiota–immune alterations may vary across disease stages. During the metabolic risk stage, early functional shifts in the microbiota and increased vulnerability of the intestinal barrier are prominent. During insulin resistance development, enhanced exposure to pro-inflammatory microbial signals and immune activation within metabolic tissues become increasingly evident. Following the onset of established T2DM, microbial functional abnormalities interact with persistent hyperglycemia, lipotoxicity, and β-cell stress in a mutually reinforcing manner. During chronic disease progression and complication stages, microbial alterations are further influenced by declining organ function, medication exposure, and long-term metabolic disturbances. It should be emphasized that this stage-based framework is primarily derived from integrated evidence from cross-sectional clinical studies, comparisons among individuals at different disease stages, animal models, and mechanistic intervention experiments. Longitudinal human studies that simultaneously track microbial composition, adaptive immune phenotypes, and glucose metabolic changes within the same individuals remain limited. Therefore, the four-stage model proposed here serves as an integrative framework for organizing current evidence rather than representing a fully validated linear causal trajectory.

### 4.1 Metabolic risk stage

T2DM generally develops under prolonged metabolic stress associated with obesity, high-fat dietary patterns, sedentary lifestyles, and metabolic syndrome (Fuentes-Barría et al., 2026). During this early stage, individuals have not yet developed persistent hyperglycemia or an overt diabetic phenotype; however, nutrient excess, lipid accumulation, and low-grade inflammation may already begin to reshape the intestinal ecosystem. Several studies suggest that individuals at elevated metabolic risk exhibit early alterations in microbial composition and functional capacity compared with metabolically healthy populations, including reduced beneficial metabolic functions, increased intestinal barrier vulnerability, and enhanced microbial-associated inflammatory signals. Notably, some of these alterations have been detected before the clinical diagnosis of diabetes in certain populations (Wu et al., 2026; Castillo et al., 2025).

Animal models of high-fat diet-induced obesity further demonstrate that microbial compositional and functional alterations may precede the development of overt hyperglycemia and are accompanied by impaired intestinal barrier integrity, increased circulating microbial-associated signals, and enhanced inflammation in metabolic tissues (Cao et al., 2026; Peng et al., 2025). Concurrently, immune tolerance mediated by Tregs may become impaired, accompanied by increased pro-inflammatory responses involving Th1, Th17, and CD8^+^ T cells, as well as dysregulated B-cell and IgA-mediated mucosal immune regulation (Dong et al., 2026; Asayesh et al., 2026; Makiel, 2026). Therefore, this stage may represent an early state characterized by microbial susceptibility and reduced immune tolerance rather than a state of established and severe microbial dysbiosis.

Mechanistically, reduced protective microbial-derived metabolic signals may weaken the capacity of Tregs to maintain immune tolerance and tissue repair, whereas increased exposure to pro-inflammatory microbial structural components and dysregulated metabolites may promote recruitment and activation of inflammatory T cells within metabolic tissues (Chen H. et al., 2026; Méndez-García et al., 2025; Shoelson et al., 2006; Liu Y. et al., 2026). However, whether these microbial and immune alterations truly precede the onset of insulin resistance in humans remains uncertain. Current evidence is largely derived from animal studies, observations in high-risk populations, and comparisons across disease stages, and the precise temporal sequence requires confirmation through longitudinal human investigations.

### 4.2 Insulin resistance stage

With persistent metabolic stress, microbial and immune abnormalities may progress from early functional deviations toward sustained dysregulation closely associated with insulin resistance. Compared with the metabolic risk stage, this phase is characterized by more prominent intestinal barrier disruption, increased exposure to pro-inflammatory microbial signals, depletion of protective microbial metabolites, and enhanced interaction between microbial alterations and inflammation in metabolic tissues (Mercado-Monroy et al., 2026; Chen K. et al., 2025).

Studies in insulin resistance models demonstrate that gut microbial dysbiosis frequently occurs alongside increased T-cell infiltration into adipose tissue, reduced Treg abundance, enhanced Th1/Th17 responses, and activation of CD8^+^ T cells (Yang H.Y. et al., 2025). Furthermore, several microbiota-targeted intervention studies have shown that restoration of intestinal microbial homeostasis may coincide with reduced metabolic tissue inflammation and improved insulin sensitivity (Li Z. et al., 2026). These findings suggest that during this stage, microbial alterations may no longer represent merely a susceptibility background but may actively participate in sustaining metabolic inflammation and insulin resistance.

Mechanistically, increased microbial structural components, dysregulated metabolites, and secreted microbial molecules may enhance antigen presentation and promote pro-inflammatory T-cell polarization, whereas inflammatory mediators produced by Th1, Th17, and CD8^+^ T cells can interfere with insulin signaling pathways in adipose tissue, liver, and skeletal muscle (Chen K. et al., 2025; Mandal et al., 2025). Nevertheless, this proposed positive feedback loop is currently supported primarily by animal and mechanistic studies. Human studies remain largely based on single-time-point observations, and the continuous causal relationship between microbial alterations, adaptive immune remodeling, and insulin resistance requires further validation through longitudinal investigations.

### 4.3 Diabetes onset stage

When persistent insulin resistance exceeds the compensatory capacity of pancreatic β cells, individuals gradually progress to established T2DM (Chen S. C. et al., 2025). Compared with the preceding stage, microbiota–immune abnormalities at this stage are superimposed on a background of sustained hyperglycemia, lipotoxicity, and β-cell stress. Accordingly, the biological significance of microbial dysregulation extends beyond the regulation of peripheral insulin sensitivity and becomes increasingly associated with systemic immunometabolic disturbance and progressive β-cell functional decline.

Patients with T2DM frequently exhibit more stable alterations in microbial functional profiles, impaired intestinal barrier integrity, and disrupted metabolite patterns, which often occur concurrently with islet inflammation, β-cell stress, and reduced insulin secretory capacity (Karthick et al., 2026). Experimental studies have suggested that modulation of the gut microbiota or supplementation with beneficial microbial-derived metabolites may improve glucose metabolism, reduce inflammation, and preserve islet function (Niu et al., 2025). In addition, tissue-based and single-cell analyses have revealed increased effector T-cell infiltration, insufficient immunoregulatory activity, and enhanced stress-associated transcriptional programs in metabolic tissues (Yuan et al., 2026).

However, studies that longitudinally follow individuals from persistent insulin resistance to the onset of T2DM while simultaneously evaluating microbial alterations, adaptive immune subsets, and β-cell function remain scarce. Therefore, whether microbiota alterations directly drive β-cell decompensation remains a mechanistically plausible hypothesis supported mainly by experimental evidence, rather than an established continuous causal pathway validated in longitudinal human studies.

### 4.4 Chronic progression and complication stage

During the chronic progression stage, persistent hyperglycemia, dysregulated lipid metabolism, oxidative stress, and low-grade inflammation continuously affect multiple organs, including the vasculature, kidneys, nervous system, liver, and cardiovascular system (Zhu L. et al., 2026). Compared with earlier stages, gut microbial alterations at this stage are more complex, as they are shaped not only by underlying metabolic abnormalities but also by declining renal function, dietary modifications, long-term medication exposure, and the complications themselves. Therefore, microbial changes observed during this stage are more difficult to interpret as primary initiating drivers of T2DM.

In diabetic complications such as diabetic kidney disease and atherosclerosis, microbial dysbiosis frequently coexists with systemic inflammation, vascular endothelial injury, and tissue fibrosis (Zhao et al., 2026; Ma et al., 2025; Blüher, 2025). Animal studies suggest that restoration of gut microbial homeostasis may partially alleviate tissue inflammation and organ injury, accompanied by enhanced immunoprotective signaling (Zou et al., 2026). Persistent exposure to microbial-derived inflammatory signals and dysregulated metabolites may sustain low-level adaptive immune activation, whereas pro-inflammatory T-cell responses and abnormal humoral immunity may further aggravate injury in vascular, renal, and other metabolic tissues. Current clinical evidence, however, remains predominantly derived from cross-sectional and case–control studies, and longitudinal investigations tracking individuals from early T2DM development to specific complications while simultaneously monitoring microbial and immune alterations remain limited.

From a therapeutic perspective, targeting the gut microbiota may theoretically provide greater opportunities for disease modification during earlier stages. During metabolic risk accumulation or early insulin resistance, intestinal barrier dysfunction, microbial functional alterations, and immune imbalance have not yet become extensively compounded by irreversible β-cell failure or established organ damage. Therefore, restoring beneficial microbial functions, metabolite production, and immune tolerance may have greater potential to interrupt subsequent pathological amplification. In contrast, after the development of established T2DM, particularly during complication stages, chronic hyperglycemia, medication exposure, and organ dysfunction become independent disease-driving factors. Under these circumstances, microbiota modulation alone is unlikely to reverse existing pathological changes and may instead serve as an adjunctive strategy within comprehensive disease management. Nevertheless, prospective randomized studies directly comparing the efficacy of microbiota-targeted interventions across different stages of T2DM are currently lacking.

## 5 Therapeutic strategies targeting gut microbiota to restore adaptive immune homeostasis

**Figure 4:**
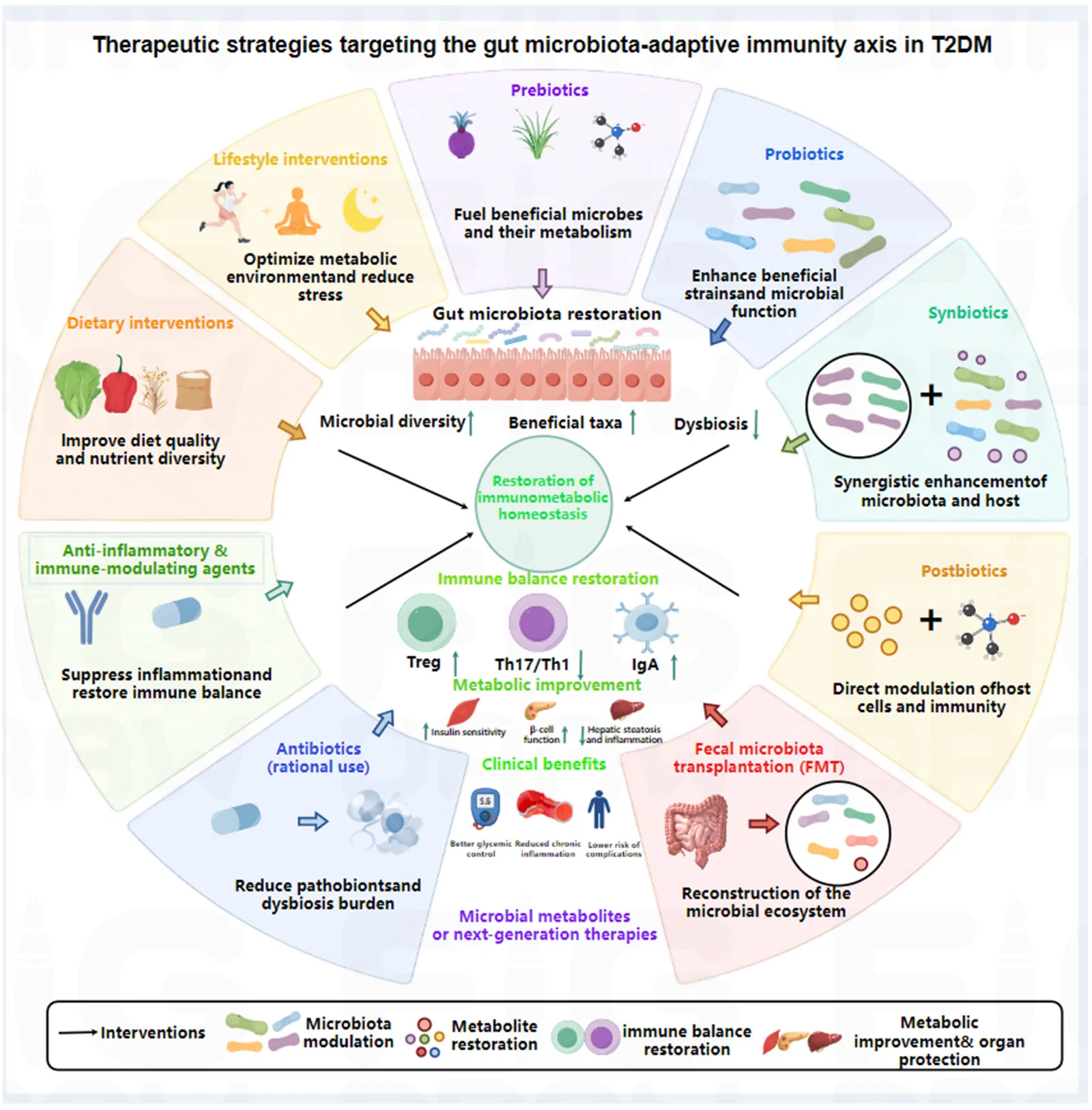
Therapeutic strategies targeting the gut microbiota–adaptive immunity axis in type 2 diabetes mellitus. Multiple microbiota-targeted interventions have been proposed to restore immunometabolic homeostasis in T2DM by reshaping microbial composition, enhancing beneficial microbial functions, and rebalancing adaptive immune responses. Dietary and lifestyle interventions improve microbial diversity and metabolic function; probiotics, prebiotics, and synbiotics promote beneficial taxa and immune tolerance; postbiotics and microbial metabolites directly modulate host immune responses; fecal microbiota transplantation reconstructs microbial ecosystems; and emerging precision approaches, including engineered microbes and phage-based therapies, aim to selectively regulate microbial functions. Through restoration of the microbiota–adaptive immunity axis, these strategies may improve insulin sensitivity, protect β-cell function, and reduce chronic inflammatory burden, although further clinical validation is required.

In recent years, therapeutic concepts for T2DM have gradually shifted from exclusive glycemic control toward improving immunometabolic abnormalities and restoring intestinal homeostasis. Microbiota-targeted interventions have expanded from conventional dietary modulation to include probiotics, prebiotics, synbiotics, postbiotics, fecal microbiota transplantation (FMT), as well as emerging approaches such as engineered microbial therapies and phage-based interventions. These strategies have demonstrated varying degrees of potential in restoring immunometabolic balance. The major mechanisms by which different microbiota-based interventions restore gut microbiota–adaptive immunity homeostasis and their potential immunometabolic benefits in T2DM are summarized in Table 2 (Mazhar et al., 2023; Hughes et al., 2022; Song et al., 2025; Ismael et al., 2021; Lauria et al., 2026; Kimble et al., 2023; Elmaleh-Sachs et al., 2023; Wang et al., 2025e; Sowah et al., 2022; Fang Y. et al., 2025; Li and Tang, 2025; Lin et al., 2025; Li G. et al., 2023; Setayesh et al., 2026; Peng et al., 2024; Li et al., 2025b; Birkeland et al., 2020; Long et al., 2025; Baroni et al., 2024; Naseri et al., 2022; Jayedi et al., 2024; Xie et al., 2025; Zhao et al., 2024; Fang H. et al., 2025; Wu et al., 2023; Gómez-Pérez et al., 2024; Zikou et al., 2024; Aminian-Dehkordi et al., 2025; Sola-Oladokun et al., 2017; Rutter et al., 2022; Kong et al., 2025; Hauza et al., 2026; Wortelboer and Herrema, 2024), with the underlying mechanisms illustrated in Figure 4.

**Table 2:** Microbiota-targeted interventions for restoring adaptive immune homeostasis and their potential immunometabolic benefits in T2DM.

Intervention category	Representative strategy	Main mechanism of action	Potential immunometabolic benefits in T2DM	Major limitations	Evidence stage	References
Dietary intervention	High-fiber diet	Increases SCFA-producing bacteria and the generation of protective metabolites such as butyrate and propionate, promotes Treg differentiation, enhances IgA-mediated barrier protection, and suppresses excessive Th17 responses	Improves insulin sensitivity, reduces metabolic endotoxemia and chronic low-grade inflammation, and alleviates adipose tissue inflammatory burden	Efficacy is influenced by fiber type, dosage, and baseline microbiota, resulting in substantial interindividual variability	RCTs/clinical studies	Niu et al., 2025; Yuan et al., 2026; Zhu L. et al., 2026
Dietary intervention	Mediterranean diet	Increases microbial diversity, enriches beneficial commensal bacteria and anti-inflammatory metabolites, enhances immune tolerance, and reduces pro-inflammatory T-cell responses	Improves glucose and lipid metabolism, reduces systemic inflammation and cardiometabolic risk, and may delay T2DM progression	Dietary protocols and adherence vary considerably, and microbiota-related outcomes remain inconsistent	RCTs/cohort studies	Zhao et al., 2026; Ma et al., 2025; Blüher, 2025
Dietary intervention	Calorie-restricted/weight-loss diet	Ameliorates obesity-associated microbial dysbiosis, reduces pro-inflammatory microbial signals, attenuates pro-inflammatory T-cell skewing, and partially restores immune homeostasis	Reduces body weight and adipose tissue inflammation and improves insulin resistance, glycemic control, and metabolic flexibility	The independent effects of microbiota modulation are difficult to distinguish from those of weight loss and caloric restriction	Clinical studies	Zou et al., 2026; Mazhar et al., 2023; Hughes et al., 2022
Lifestyle intervention	Regular exercise	Increases microbial diversity, improves SCFA-related metabolic function, reduces systemic inflammation, and promotes Treg/Th17 balance	Improves insulin sensitivity, weight management, and metabolic flexibility and reduces chronic inflammatory burden	Exercise protocols are highly heterogeneous, and microbiota changes and their independent effects remain inconsistent	Clinical studies	Song et al., 2025; Ismael et al., 2021; Lauria et al., 2026
Probiotics	*Lactobacillus, Bifidobacterium*, and related strains	Increases the abundance of beneficial bacteria, suppresses the expansion of opportunistic pathogens, improves intestinal barrier function, promotes Treg and IgA responses, and reduces Th1/Th17-associated inflammation	Reduces low-grade inflammation and gut-derived antigen exposure and may improve fasting glucose, HbA1c, and insulin resistance indices	Efficacy is strain- and population-dependent, and some RCTs have reported no significant glucose-lowering effects	RCTs/meta-analyses	Kimble et al., 2023; Elmaleh-Sachs et al., 2023; Wang et al., 2025e
Prebiotics	Inulin, fructooligosaccharides, and related substrates	Promotes the growth of *Bifidobacterium* and SCFA-producing bacteria, enhances Treg- and IgA-associated mucosal immunity, and suppresses pro-inflammatory responses	Increases protective metabolites and improves intestinal barrier function, insulin sensitivity, and glucose and lipid metabolism	Efficacy depends on baseline microbiota composition and substrate-utilization capacity, resulting in marked interindividual variability	RCTs/clinical studies	Sowah et al., 2022; Fang Y. et al., 2025; Li and Tang, 2025
Synbiotics	Combined probiotics and prebiotics	Simultaneously provides beneficial microorganisms and fermentable substrates, enhances microbial functional output, promotes immune tolerance, and reduces pro-inflammatory T-cell responses	Improves glycemic control, insulin resistance, and inflammatory status and may enhance the effects of probiotics or prebiotics alone	Formulations vary substantially, long-term evidence is limited, and consistent superiority over single-component preparations has not been established	RCTs/meta-analyses	Lin et al., 2025; Li G. et al., 2023; Setayesh et al., 2026
Postbiotics	Butyrate, inactivated microbial cells, and microbial components	Directly supplies microbial functional products or immunoregulatory components, induces Foxp3^+^ Tregs, and enhances barrier protection and IgA responses	Reduces metabolic endotoxemia and improves intestinal barrier integrity, low-grade inflammation, and insulin signaling	Human evidence is limited, and active components, dosing, and manufacturing standards remain insufficiently standardized	Animal studies/early clinical exploration	Peng et al., 2024; Li et al., 2025b; Birkeland et al., 2020
Microbiota transplantation	FMT from healthy donors	Reconstructs microbial diversity and metabolic function, restores commensal microbial networks, and may improve Treg/Th17 balance and immune tolerance	May improve insulin sensitivity and microbial metabolic function, although responses are influenced by donor and recipient characteristics, dietary background, and baseline microbiota	Strong donor–recipient dependency, uncertain durability and long-term safety, and insufficient standardization	Exploratory clinical studies	Long et al., 2025; Baroni et al., 2024; Naseri et al., 2022
Engineered bacteria/live biotherapeutic products	Engineered probiotics and live biotherapeutic products	Delivers SCFAs, anti-inflammatory molecules, or barrier-protective factors in a targeted manner, thereby enhancing Treg, IgA, and other anti-inflammatory immune responses	May precisely modulate the microbiota–adaptive immunity axis and improve inflammation and metabolic abnormalities, although safety and stability require further validation	Evidence is predominantly preclinical, and stability, biosafety, and regulatory challenges remain unresolved	Preclinical/early-stage studies	Jayedi et al., 2024; Xie et al., 2025; Zhao et al., 2024
Bacteriophage therapy	Bacteriophages targeting opportunistic pathogens	Selectively eliminates specific pro-inflammatory bacteria, reduces microbial antigens, virulence factors, and pro-inflammatory metabolite burden, and indirectly restores immune homeostasis	May reduce gut-derived inflammatory stimulation and improve microbial ecological niches and insulin resistance-associated inflammation, although direct evidence in T2DM is limited	Direct evidence in T2DM is extremely limited, and host range, formulation stability, and regulatory issues remain major constraints	Experimental studies	Fang H. et al., 2025; Wu et al., 2023; Gómez-Pérez et al., 2024

### 5.1 Dietary and lifestyle interventions

Dietary and lifestyle interventions influence gut microbial ecology by modifying nutrient availability, energy balance, and overall metabolic status, representing the most fundamental approaches for T2DM management. High-fiber diets increase fermentable substrates available to intestinal microorganisms, promoting the expansion of SCFA-producing bacteria and the generation of beneficial metabolites such as butyrate and propionate. These microbial-derived metabolites support Treg differentiation, IgA-associated barrier protection, and immune tolerance. Mediterranean diets, characterized by increased intake of whole grains, vegetables, fruits, legumes, and unsaturated fatty acids, enhance microbial diversity and beneficial metabolite production and have relatively strong human evidence supporting improvements in glucose and lipid metabolism as well as systemic inflammation. Energy-restricted diets, weight reduction, and regular physical activity can also improve metabolic status and are accompanied by alterations in microbial function and immune homeostasis.

Different dietary patterns exhibit distinct biological characteristics and clinical effects. Whole-food plant-based diets, which are rich in dietary fiber and phytochemicals, have recently been shown in randomized controlled studies to improve glycemic control in individuals with T2DM and reduce the need for glucose-lowering medications in some patients when combined with lifestyle interventions (Hanick et al., 2025). In contrast, very-low-carbohydrate or ketogenic diets may provide weight loss and short-term metabolic benefits in selected individuals; however, their long-term superiority in HbA1c reduction remains inconsistent during 2-year follow-up (Strombotne et al., 2024). Moreover, their lower dietary fiber content may produce microbiome effects that differ from those induced by Mediterranean or plant-based dietary patterns. Therefore, current evidence does not support the conclusion that any single dietary pattern is universally superior based solely on its microbiota-modulating properties.

Patient adherence and long-term sustainability are equally critical determinants of real-world effectiveness. Strict energy restriction or very-low-carbohydrate diets may be difficult to maintain over extended periods, whereas Mediterranean diets generally demonstrate better sustainability but remain influenced by dietary preferences, social environments, and individual variability. Accordingly, dietary modification and physical activity should remain foundational components of long-term T2DM management. Future strategies should integrate baseline microbial characteristics, patient adherence, and long-term metabolic outcomes to develop individualized dietary stratification approaches.

### 5.2 Microbiota-based interventions

Microbiota-based interventions, including probiotics, prebiotics, synbiotics, and postbiotics, may influence immunometabolic homeostasis in T2DM by enhancing beneficial metabolite production, improving intestinal barrier integrity, reducing microbial antigen exposure, and modulating Tregs, Th1, Th17, B-cell, and IgA-mediated immune responses. Current randomized controlled trials and meta-analyses suggest that certain probiotics, prebiotics, and synbiotics may modestly improve fasting glucose, HbA1c, insulin resistance, and inflammatory markers; however, the findings remain inconsistent across studies.

The effects of probiotics are highly strain-specific and cannot be generalized based solely on bacterial genus-level classification, such as Lactobacillus or Bifidobacterium. A recent randomized double-blind controlled trial in individuals with T2DM evaluated a combined probiotic intervention containing Bifidobacterium animalis subsp. lactis BLa80, Lacticaseibacillus rhamnosus LRa05, and Bifidobacterium breve BBr60. The intervention was associated with reduced IL-17 and TNF-α levels, accompanied by alterations in bile acids, SCFAs, microbial composition, and selected glycemic parameters (Zhu S. et al., 2026). However, these findings cannot be directly extrapolated to other strains within the same genus or species.

The inconsistency among probiotic studies may be attributable to differences in bacterial strains, dosage, intervention duration, formulation stability, baseline microbiota composition, dietary background, obesity status, and concomitant glucose-lowering medications. Moreover, clinical endpoints vary substantially among studies. Therefore, probiotics currently do not demonstrate stable or predictable glucose-lowering efficacy. Regarding safety, most studies in T2DM populations indicate that probiotics and synbiotics are generally well tolerated, with adverse events mainly limited to mild gastrointestinal symptoms. Nevertheless, the use of live microbial products requires caution in individuals with severe immune dysfunction or marked intestinal barrier disruption. The efficacy of prebiotics depends largely on the baseline microbial ecosystem, synbiotics lack standardized combinations, and postbiotics continue to face challenges related to active components, dosing strategies, and quality control. Overall, microbiota-based supplements are currently better positioned as adjunctive approaches to standard glucose-lowering therapies rather than independent treatments for T2DM.

### 5.3 Microbiota restoration and emerging microbiome-based therapies

FMT, engineered bacteria, and phage-based therapies represent more active and targeted approaches for microbiome modulation; however, their clinical maturity varies substantially. FMT has been explored in individuals with T2DM and severe insulin resistance in early clinical studies. Some investigations suggest that FMT may improve microbial composition, functional capacity, and insulin sensitivity. However, therapeutic efficacy is strongly influenced by multiple factors, including donor characteristics, recipient baseline microbiota, administration route, dietary patterns, and medication exposure. Long-term microbial engraftment and sustained clinical benefits remain uncertain. Safety and regulatory issues also represent major barriers to clinical translation. Donors may carry asymptomatic pathogens or multidrug-resistant organisms, necessitating rigorous screening procedures for medical history, infectious risks, and antimicrobial resistance profiles (Ng et al., 2024). Regulatory frameworks regarding FMT product classification, manufacturing standards, and clinical applications remain heterogeneous across different countries and regions (Hoffmann et al., 2025). In addition, ethical considerations involving informed consent, donor privacy, and unknown long-term risks require further attention. Therefore, FMT should currently be considered an investigational intervention rather than an established therapy for T2DM.

Engineered bacteria and live biotherapeutic products (LBPs) represent a rapidly evolving field in which synthetic biology approaches are used to introduce specific metabolic or therapeutic functions into selected microbial strains. This area has begun to enter early-phase human studies. A Phase 1/2a clinical study published in 2025 demonstrated dose-dependent and reversible intestinal colonization of engineered *Phocaeicola vulgatus* in humans, accompanied by functional metabolic outputs, while also highlighting translational challenges such as genetic stability (Whitaker et al., 2025). Importantly, this study was not designed for T2DM, and current evidence supporting engineered bacteria for improving glycemic control or clinical outcomes in T2DM remains largely restricted to preclinical investigations.

Phage therapy offers a relatively precise strategy to eliminate specific microbial populations associated with inflammatory phenotypes. However, direct evidence in T2DM remains extremely limited. Challenges including host specificity, phage resistance, ecological consequences of microbial niche alteration, formulation stability, and regulatory uncertainty continue to restrict clinical application. Therefore, according to the current evidence hierarchy, dietary interventions and selected microbiota-based supplements have obtained support from randomized controlled trials and meta-analyses; FMT has generated limited exploratory human evidence; engineered bacteria have entered early clinical evaluation in other metabolic contexts but remain largely preclinical for T2DM; and phage-based therapies are still primarily supported by experimental studies.

## 6 Conclusions and future perspectives

The development and progression of T2DM are not solely driven by disturbances in glucose and lipid metabolism but are also shaped by continuous interactions between gut microbial alterations and adaptive immune dysregulation. Increasing evidence indicates that microbial structural components, metabolites, and other microbiota-associated signals participate in chronic low-grade inflammation, insulin resistance, β-cell functional decline, and diabetes-associated complications through regulation of T-cell responses, B-cell function, and IgA-mediated mucosal immunity. However, current evidence remains largely derived from cross-sectional clinical studies, animal models, and *in vitro* experiments. Substantial heterogeneity exists among studies due to differences in population characteristics, disease stages, dietary patterns, medication exposure, and analytical methodologies. In particular, whether microbial alterations represent initiating drivers of T2DM progression, secondary consequences of metabolic disturbances, or outcomes of reciprocal interactions remains unresolved. Establishing reliable temporal relationships and causal mechanisms therefore represents one of the major challenges in this field.

Future studies should prioritize longitudinal, multicenter, and geographically diverse cohorts to continuously monitor microbial, immune, and metabolic changes from metabolic risk states and insulin resistance to established T2DM and its complications. Integrative approaches combining metagenomics, metatranscriptomics, metabolomics, immune profiling, and clinical metabolic parameters will be essential to move beyond descriptive characterization of microbial composition toward understanding dynamic relationships among microbial functions, metabolic outputs, and adaptive immune responses. Advanced experimental platforms, including intestinal organoids, immune cell co-culture systems, and organ-on-chip models, may further facilitate causal validation of candidate microorganisms, metabolites, and immune pathways, thereby bridging the gap between associative observations and mechanistic understanding.

From a translational perspective, future microbiome-targeted intervention trials should adopt standardized, multicenter, randomized controlled designs and incorporate predefined stratification based on disease stage, baseline microbiota characteristics, dietary patterns, medication exposure, and immune phenotypes. Clinical outcomes should integrate microbial engraftment or functional alterations, immunological parameters, metabolic endpoints such as HbA1c and insulin resistance, as well as long-term safety and complication outcomes. For FMT, engineered bacteria, and other emerging microbiome-based therapies, extended follow-up will be essential to evaluate durability of therapeutic effects, ecological stability, and potential delayed risks. Artificial intelligence and machine learning approaches may further integrate microbiome composition, metabolomic profiles, transcriptomic signatures, immune phenotypes, and clinical characteristics to identify complex microbiome–immune signatures, predict therapeutic responses, and define patient populations most likely to benefit. Ultimately, only through the integration of longitudinal causal studies, multi-omics mechanistic investigations, and stratified clinical trials can interventions targeting the gut microbiota–adaptive immunity axis advance from associative observations toward precision medicine approaches for T2DM.
